# Recent Advances in Amorphous Solid Dispersions: Preformulation, Formulation Strategies, Technological Advancements and Characterization

**DOI:** 10.3390/pharmaceutics14102203

**Published:** 2022-10-16

**Authors:** Srushti Tambe, Divya Jain, Sai Kishore Meruva, Gopinath Rongala, Abhishek Juluri, Girish Nihalani, Hemanth Kumar Mamidi, Pavan Kumar Nukala, Pradeep Kumar Bolla

**Affiliations:** 1 Department of Pharmaceutical Science and Technology, Institute of Chemical Technology, Mumbai 400019, India; 2 College of Pharmacy, University of Iowa, Iowa City, IA 52242, USA; 3 Venatorx Pharmaceuticals, Inc., 74 E Swedesford Rd. Suite 100, Malvern, PA 19355, USA; 4 Department of Pharmaceutics, The University of Mississippi, Oxford, MS 38677, USA; 5 Teva Pharmaceuticals USA, Weston, FL 33331, USA; 6 College of Pharmacy and Health Sciences, St. John’s University, Queens, NY 11439, USA; 7 Department of Biomedical Engineering, College of Engineering, The University of Texas at El Paso, 500 W. University Ave., El Paso, TX 79968, USA

**Keywords:** amorphous solid dispersions, glass transition temperature, poorly soluble drugs, super saturation, recrystallization, surfactants, lipids, pill burden, spray drying, hot melt extrusion, Kinetisol^®^, 3D printing, characterization, polymers

## Abstract

Amorphous solid dispersions (ASDs) are among the most popular and widely studied solubility enhancement techniques. Since their inception in the early 1960s, the formulation development of ASDs has undergone tremendous progress. For instance, the method of preparing ASDs evolved from solvent-based approaches to solvent-free methods such as hot melt extrusion and Kinetisol^®^. The formulation approaches have advanced from employing a single polymeric carrier to multiple carriers with plasticizers to improve the stability and performance of ASDs. Major excipient manufacturers recognized the potential of ASDs and began introducing specialty excipients ideal for formulating ASDs. In addition to traditional techniques such as differential scanning calorimeter (DSC) and X-ray crystallography, recent innovations such as nano-tomography, transmission electron microscopy (TEM), atomic force microscopy (AFM), and X-ray microscopy support a better understanding of the microstructure of ASDs. The purpose of this review is to highlight the recent advancements in the field of ASDs with respect to formulation approaches, methods of preparation, and advanced characterization techniques

## 1. Introduction

Aqueous solubility and permeability across biological membranes are essential prerequisites for effective oral absorption [1]. Around 70–90% of all new chemical entities (NCEs)/drug molecules under development were reported to possess poor aqueous solubility; hence, they belong to the Biopharmaceutics Classification System (BCS) class II or class IV drugs [2,3]. This phenomenon of poor solubility is due to the structure and functional groups identified during the drug discovery phase. In an attempt to improve the poor solubility of NCEs, approaches such as modifying the structure–activity relationship (SAR) were tried during the preclinical development stage [4]. However, the use of these approaches on NCEs is often limited, due to the requirement of a lipophilic nature to bind biological targets or to cross biological membranes [5]. Therefore, this resulted in an increased number of poorly aqueous soluble NCEs in the preclinical development, posing a challenge during formulation development [6]. Based on physicochemical properties such as melting point, logP, molecular weight and aqueous solubility, NCEs were classified as either ‘brick-dust’ molecules or ‘grease-ball’ molecules [7,8]. The former name indicates solid-state limited solubility (due to orderly arranged crystalline lattices) and the latter denotes solvation-limited solubility (due to high lipophilicity) [9]. Therefore, as mentioned earlier, aqueous solubility and membrane permeability were found to be pivotal in the pipeline of successful formulation development. Depending on the inherent solubility and permeability of drug molecules, researchers have explored various formulation approaches for improvement [10]. Based on the methods reported in the literature, lipid-based drug delivery, micronization, use of polymorphs, co-crystals, salt formation, prodrug, nanocrystal dispersion, cyclodextrin complexation, binding to ion exchange resins, and amorphization were found to be effective [11,12,13,14]. Among these solubility enhancement technologies, amorphous solid dispersions (ASDs) have attracted tremendous importance in the last decade with numerous marketed products. Sekiguchi and Obi [15] first proposed the concept of solid dispersions in 1961. By definition, in ASDs, the drug homogenously disperses in an excipient carrier in amorphous state. The amorphous form of API enhances solubility by lacking crystalline lattices and having an inherently disordered arrangement. Apart from improving the solubility, ASDs enhance the wettability, rate of dissolution, and supersaturation of drugs, thereby promoting the membrane flux, ultimately leading to improved oral bioavailability [16]. The combination of a rapidly dissolving and supersaturating “spring” with precipitation retarding “parachute” is employed as an efficient formulation strategy for ASDs to improve the rate and extent of oral absorption [17]. Since then, solid dispersion (SD) technology has attracted the scientific community, leading to extensive research in the field of ASDs. At this time, nearly >25 ASD formulations are commercially available on the market [18]. Table 1 summarizes the Food and Drug Administration (FDA)-approved ASDs along with the polymers used in the formulation.

However, ASDs are susceptible to thermodynamic instability (conversion from an amorphous state to a crystalline state) due to the higher free energy associated with the amorphous state [19,20]. Numerous factors such as the improper selection of formulation components, thermal stress, environmental stress (humidity), and manufacturing stress contribute to the physical instability of ASD. The proper selection of formulation ingredients, manufacturing process, process parameters, and packaging components are deemed essential to obtain a stable ASD drug product [21].

Since their introduction, the formulation and development of ASDs has come a long way from the simple conversion of crystalline drugs to an amorphous form devoid of polymers to the inclusion of polymers and surfactants for hindering recrystallization and maintaining supersaturation [22]. Therefore, over the decades, ASDs have seen various advances in development, which include, but are not limited to, formulation development techniques (use of polymers, salts, and surfactants), manufacturing techniques, and characterization techniques [23,24]. Broadly, ASDs can be classified into three generations, which include amorphous forms of drugs (first generation), use of polymeric carriers (second generation), and the use of amorphous carriers and surfactants (third generation) [25]. The first generation of ASDs was formulated by converting crystalline drug molecules into amorphous forms through solvent evaporation technique without any polymeric carriers. Since this approach is limited to a lower number of drug molecules, attention was shifted to second-generation ASDs, in which polymeric carriers are included to stabilize the amorphous drug molecule [26]. Although this approach expanded the horizon of ASDs to formulate several drug molecules with poor solubility, a few second-generation ASDs suffered from issues such as recrystallization and the inability to maintain supersaturation during dissolution. This led to the development of third-generation ASDs, where surfactants were included along with polymeric carriers to maintain supersaturation and achieve targeted bioavailability [27]. Going forward, to meet the current requirements of ASD formulations, approaches such as the alteration/improvisation of existing techniques, use of novel techniques, and advances in characterization techniques for ASDs evolved [28]. Therefore, traditional approaches such as solvent evaporation and melt cooling were updated to be better suited to commercialization. Consequently, the existing technologies prevailed to spray-dryers and hot melt extruders, making them viable for ASD commercial manufacturing [10]. Additionally, the developments in the manufacturing arena helped to identify the impact of process variables over critical quality attributes (CQA’s) of ASDs and also led to the development of novel manufacturing processes like Kinetisol^®^ dispersing (KSD) [29], selective laser sintering (SLS) [30] and fused deposition modeling (FDM) 3D printing [31], electronanospinning, and others [32]. It is worth mentioning the recent advancements in preformulation space, which aimed to understand the interactions between the formulation components at a molecular level [33]; this was possible through analytical techniques and molecular simulation predictions using in-silico tools [34]. Besides the advances in formulation and preformulation approaches, traditional analytical techniques such as differential scanning calorimetry (DSC) and powder X-ray diffraction (PXRD) still play a major role in detecting crystallinity [35]. However, in recent times, traditional techniques supplemented with advanced imaging technologies such as nanotomography, tetrahertz spectroscopy and others [36,37,38,39] helped to characterize ASDs at submicron levels determining the intermolecular interaction between the amorphous drug and polymeric carrier. The added advantage of novel characterization techniques lies in their ability to predict dissolution behavior, recrystallization mechanism and stability performance [40,41,42,43].

In view of the recent advances in ASDs formulation development, the present review focuses on recent advances with respect to preformulation screening, formulation approaches, manufacturing techniques, and characterization techniques.

## 2. Recent Advances in Preformulation

The two main aspects of preformulation assessment are saturation solubility and stability, which inherently depend on active pharmaceutical ingredient’s (API’s) properties and the added excipients used in the formulation. A tremendous amount of research has been conducted in the past few decades, which mainly emphasized the amorphous advantage of APIs, the selection of proper excipients, and the prediction of the physical stability of ASDs. In the past, traditional methods employed a large amount of API material to determine these properties. Considering recent advances, several material-sparing methods were developed for initial preformulation assessment [44,45]. As specified in the available literature, the difference in free energy is one of the notable properties that helps to understand the advantage of solubility for the amorphous form over the crystalline form. The higher the free energy of the amorphous form, the better its solubility. Research progression in this field led to the development of DSC as the easiest and quickest method to determine the free energy difference between the crystalline and amorphous API using the following equation (Equation (1)) [46,47,48].

(1) $$\Delta G=-RTln\frac{a_{amorphous}}{a_{crystalline}}$$

where Δ*G* is the free energy difference between crystalline and amorphous form, R is the real gas constant, ‘*a*’ is the activity coefficient of the amorphous or crystalline form in the solution. The most straightforward approach to the determination of the activity co-efficient difference between amorphous and crystalline forms is to determine the difference in heat capacity values (ΔCp) between the two forms using modulated DSC (MDSC) [49].

The free energy difference obtained using this technique could help in understanding the ability of the amorphous form relative to crystalline form to overcome issues such as poor in vivo exposure. Although this exists as a well-established method in solubility estimation, the role of DSC in preformulation screening remains a promising tool for determining the API and polymer proportions of many NCEs.

Furthermore, after selecting the right API and polymer candidate (in definite proportions) based on free energy difference, further calculations will be performed based on solute activity and a fraction of ionized species. In the case of ASDs prepared using hot melt extrusion (HME), the ideal processing conditions, such as drug loading and processing temperatures, can be determined using DSC-based material sparing method proposed by Mamidi and Rohera [50]. This approach requires a few grams of API and an ideal preformulation technique to determine if melt-based techniques are needed for certain APIs.

Another important aspect in the preformulation assessment of ASDs includes physical stability, which can eventually lead to recrystallization in unstable formulations. Therefore, APIs have been categorized into three classes (I, II, and III) that determine their glass-forming ability (GFA) based on the recrystallization potential of API, which is obtained during the heating–cooling and reheating cycle performed in DSC [51]. If the API recrystallizes during the cooling cycle, it is categorized as GFA Class I, and if it recrystallizes during the reheating cycle, it is categorized as GFA Class II. GFA class III APIs do not recrystallize during the cooling or reheating cycle. Among these three classes, class III APIs are the most preferred forms for the formulation of ASDs [52]. In addition to the above methods, determining the ratio of melting point/glass transition temperature (T_m_/T_g_) is used to assess the amorphous stability of the API. The higher the T_m_/T_g_ ratio, the higher the tendency of API to recrystallize. Therefore, APIs with T_m_/T_g_ ratios on the lower side are desirable for the formulation of ASDs [53]. Since the stability of ASDs also depends on external factors such as moisture uptake (hygroscopicity) and storage temperature, several theoretical approaches including ternary or quaternary phase models (Flory–Huggin’s theory [54,55], Perturbed-Chain Statistical Associating Fluid Theory, and other hybrid models) have been proposed in the literature [56].

Once the assessment of API is completed, the next step is to select the appropriate polymer for the formulation of ASDs. This critical step involves both the selection of the ideal polymers and their composition in the final formulation. The traditional approach for the selection of the right polymer mainly involves trial and error, accompanied by DSC to ensure a homogeneous drug–polymer mixture. Lately, the field of ASDs has involved the use of the COSMOtherm approach to determine the intermolecular interactions between the drug and polymer [49]. This is an API-free technique and a completely in silico approach. It is based on computer simulations involving polar groups and H-bonding sites. The in silico approach relies on predicting the miscibility of groups when projected in a three-dimensional spatial arrangement. Once the in silico prediction results are available, this is followed by conducting small-scale experiments to validate the miscibility between API, polymer, and any added surfactant in well-defined proportions. Miscibility experiments were carried out by film-casting technique coupled with characterization techniques such as polarized light microscopy (PLM), DSC, and PXRD.

## 3. Advances in ASD Formulation Approaches

Despite tremendous improvements in formulation approaches, most of the marketed ASD formulations are commercially manufactured using hot melt extrusion and spray drying [57]. Therefore, before discussing the advances in formulation development involving these technologies, we have attempted to briefly discuss the actual process.

The HME method involves pumping the drug(s) and excipients into an extruder with revolving screw elements at temperatures typically greater than the glass transition temperature (T_g_) of polymers and, in some cases, considerably higher than the melting temperature (T_m_). Inside the heated barrel, the melting of drug and excipients take place with distributive and dispersive mixing to enhance the molecular mixing [58,59]. This process leads to the production of extrudates/filaments with superior content uniformity and higher quality [60]. Further, these extrudes are milled, blended, and encapsulated into capsules or compressed into tablets [61,62,63,64]. HME has emerged as a ground-breaking manufacturing technology in the pharmaceutical industry, and its use is expanding as a result of its continuous production, solvent less process, affordable, industrially feasible, highly reproducible results, automation potentiality, and real-time monitoring. However, for smooth extrusion, the process temperature must generally be greater than the T_m_ or T_g_ of the carrier, this will allow for adequate flow through the extruder. As a result, the T_m_ or T_g_ of the carrier should not be too high to reduce the risk of drug degradation and/or to provide a temperature that can be used effectively. This is one of the drawbacks of HME technology in the case of carriers/polymers having higher T_m_ or T_g_ and extruding thermally labile drugs. At present, industries are developing excipients specific for use in HME, and with the aid of a plasticizer, the processing temperature can be lowered.

Spray-drying is a well-known method for the preparation of ASDs. In this process, the drug and polymer are initially dissolved in a common organic solvent. Later, this solution is pumped through a nozzle and atomized into fine droplets. These fine droplets are passed into a drying chamber followed by the wet gas, and the resultant dry powder particles are separated in a cyclone chamber. Finally, the product is deposited into the collecting vessel [65,66,67,68,69]. Spray-drying is a robust manufacturing process that produces ASDs at a commercial scale. This process is suitable for the amorphization of thermolabile drugs. However, the drying process is required since this industrial process uses solvents. Due to the above short-coming, there might be residual solvent present in the final product, which should be monitored and regulated according to International Council for Harmonisation of Technical Requirements for Pharmaceuticals for Human Use (ICH) guidelines [70].

### 3.1. Mitigating the Pill Burden by High Drug Loaded ASDs

Based on a database from FDA, more than 20 ASD drugs were approved; among them, more than 50% have doses higher than 100 mg [18,27]. Between 2015 and 2020, approximately 14 new ASDs were approved [71]. In a process of formulating a stable, a robust ASD product of higher strength, would require a higher amount and number of excipients. During this process, the dosage form may end up having a higher mass, which, in turn, can affect its dimensions in some cases. Therefore, the high mass of oral formulations cannot be amenable to geriatric populations for palatability.

To attenuate the current issue, various researchers have worked to develop stable and robust high drug-loaded ASD, with a primary goal of reducing pill burden and tablet mass. Interestingly, the thermal and viscoelastic properties of hydrophilic carriers such as glass transition melt viscosity and chemical substitutions on the carrier and molecular weight were researched to serve this purpose.

In a similar study, Mudie et al. [72] worked on a low T_g_ (42 °C) highly crystalline, poorly soluble (BCS class II), rapidly crystallizing drug erlotinib. This is a drug of choice in pancreatic and non-small cell lung cancer at a dose of 100 and 150 mg per day, respectively. They described the art of manufacturing high-loaded dosage form approach (HLDF). In this approach, a high-T_g_ polymer was spray-dried with API to obtain ASD. The resultant ASDs were granulated using high-T_g_ cellulose-based pH-dependent polymer (concentration sustaining polymer (CSP) to maintain supersaturation at higher drug load, later tablets were compressed using the final mixture. In brief, spray-dried dispersions (SDD’s) were made using 65% *w*/*w* drug load with Eudragit L100 (T_g_ 150 °C), which was followed by granulation with 29% *w*/*w* of HPMCAS H and blending with lactose and cab-o-sil prior to compression. Using the HLDF technique, the authors have successfully demonstrated a tablet formulation with 350 mg mass and a drug loading of 29% *w*/*w*, relative to traditional formulation having a tablet weight of 575 mg with 18% *w*/*w* drug loading. The formulation with a higher drug load remained stable without recrystallization after subjecting to accelerated stability conditions (40 °C ± 2 °C/75% RH ± 5% RH) for one week. Additionally, the in vitro dissolution data between HLDF and traditional formulations, respectively were highly comparable. Therefore, this study proclaimed the importance of novel HLDF technology in reducing the tablet mass by 40% without comprising physical stability and in vitro release.

Mudie et al. also investigated the HLDF technology on another poorly soluble anti-fungal drug, Posaconazole, a low-T_g_ (59 °C) and poorly soluble drug (BCS class II). By employing this technology, the authors were able to formulate low-mass tablets with a high drug load of 40% *w*/*w* without any recrystallization. Thus, this could reduce the tablet mass by 40% relative to the conventional Posaconazole formulation. The HLDF tablet was even able to outperform the conventional formulation in the in vivo study conducted on beagle dogs [73].

AstraZeneca marketed a capsule dosage form, Lynparza (Olaparib) 50 mg strength for treating women with germline BRCA recurrent advanced ovarian cancer. This formulation was approved as a capsule dosage form by FDA in December 2014. This formulation contains BCS class IV poorly soluble drug Olaparib, having a dosage regimen of 800 mg per day. Therefore, the patient used to consume approximately 16 capsules a day (8 capsules/400 mg twice daily). Initial capsule formulation of 50 mg strength has been discontinued, which utilized lipids (Gelucire 44/14) for solubility and bioavailability enhancement. In order to provide a patient compliant convenient therapy, Bechtold et al., from AstraZeneca, reformulated the product by developing a novel ASD formulation using HME to cut down the pill burden. The researchers investigated a wide range of polymers and surfactants in their study. The final formulation was developed using a 25% *w*/*w* drug load with co-povidone (PVP/VA64) and, subsequently, the extrudates were milled and compressed into tablets with the addition of excipients. Successfully, 100 mg and 150 mg strength tablets were developed and introduced into the market, after FDA approval in August 2017. With this approach, the pill burden was convenient and reduced to 300 mg twice daily or2-4 tablets per day depending on personalized treatment based on prevailing medical condition [74].

Along these lines, various advances have taken place in the way ASDs are formulated, with the main objective of increasing the drug load and maintaining supersaturation. This includes patented SUBA^TM^ technology, the use of polymeric carrier combinations, the use of surfactants, and the chemical modification of polymers into polymeric salts. A detailed description of these approaches is discussed in the following section, along with some case studies.

### 3.2. SUBA™ Technology (via Spray Drying Process)

The oral bioavailability of poorly soluble active ingredients is enhanced by the patented SUBA^TM^ technology, with superior bioavailability. Initially, Mayne Pharma USA developed this technology. The main objective behind developing this technology relies on improving bioavailability, reduction in dose, mitigating inter and intra-patient variation, enabling more predictive clinical response based on dose, and obtaining clinical levels of activity in the blood stream [75].

For the very first time, this process was used on the well-known, poorly soluble compound BCS class II compound Itraconazole. The FDA-approved prescription drug Itraconazole has a long history of safe and successful usage in treating severe fungal or yeast infections in people. The novel SUBA^TM^ process produces amorphous Itraconazole dispersed in a polymer matrix instead of a conventional crystalline form, marketed as TOLSURA^®^ in the US.

This approach utilizes spray-drying with enteric polymer to increase active ingredient solubility in the gastrointestinal system to achieve “super bioavailability” in comparison to traditional formulations. In this technology, API was spray-dried using a novel amorphous pH-dependent enteric polymer HPMC Phthalate. Unlike conventional Itraconazole, TOLSURA^®^ is insoluble in the acidic environment of the stomach and soluble in the higher pH of the small intestine [76]. The large surface area of the small intestine and nanosized particles of SUBA^TM^ Itraconazole improve bioavailability and reduce patient variability. The oral solution and capsule forms of conventional Itraconazole exhibit variable pharmacokinetics due to inconsistent absorption. Therefore, they can cause up to 15-fold interpatient heterogeneity; additionally, their absorption is impacted by the presence of diet. As a result, Itraconazole and its main active metabolite, hydroxyitraconazole, might have unanticipated supratherapeutic or sub-therapeutic plasma levels. Hence, the developed new SUBA^TM^-Itraconazole has a relative bioavailability of 180 percent and an absolute bioavailability of up to 90 percent in comparison to conventional preparation [77]. SUBA^TM^-Itraconazole has increased absorption and greatly decreased variability, giving patients and prescribers a more predictable clinical dosage response, and lowering the amount of active medication needed. With fewer side effects, the 65 mg capsule SUBA^TM^-Itraconazole formulation achieves bioequivalence to a 100 mg capsule of conventional Itraconazole.

### 3.3. Use of Polymeric Combinations for Enhanced Bioavailability

#### 3.3.1. Polymeric Combinations in HME

In the case of poorly soluble drugs, better stabilization and prolonged supersaturation is highly essential for desired or sufficient drug release. Generally, the inclusion of a hydrophobic polymer with a poorly soluble drug can lead to prolonged release and extended supersaturation by means of, a. intermolecular hydrogen bonding and b. hydrophobic interactions between API and polymer. Conversely, the addition of a hydrophilic polymer to a poorly soluble drug results in rapid drug release; however, in later stages, it can eventually lead to nucleation and precipitation. Therefore, few researchers investigated a combination of hydrophilic and hydrophobic polymers to achieve rapid dissolution associated with prolonged supersaturation. In a similar line, Butreddy et al. [78] developed ASD of Nifedipine (30% *w*/*w* DL) by HME using various combinations of hydrophilic (PVP, co-povidone) and hydrophobic polymers (HPMC AS HG, Eudragit RSPO, Eudragit FS 100) with HPMC AS LG. The researchers focused on transitions involving the solid state of API, drug release, the particle size of supersaturated solution after a prolonged time (to evaluate inhibition of nucleation), non-sink dissolution in pH 6.8 buffer and dissolution profiles of stability samples from 40 °C/75% RH. The author’s findings explained that a combination of 50% *w*/*w* HPMC-AS L and 20% *w*/*w* HPMC-AS H outperformed the other polymeric mixtures. Their key findings included (a) conversion of API to amorphous; (b) extended API super-saturation due to the inclusion of H-grade (20% *w*/*w*) with L-grade (50% *w*/*w*); (c) no increase in particle size was noted after 120-min; d. lack of significant difference in non-sink dissolution profiles between initial and stability samples from the above-mentioned combination. The superior performance of this formulation in terms of release and supersaturation was based on high-succinoyl groups from hydrophilic HPMC-LG and acetyl groups from HPMC-HG, respectively [79].

Another study by Wlodarsky et al. [80] exhibited enhanced dissolution and supersaturation with ternary ASD via HME, comprising the poorly soluble drug Itraconazole, PVA, and co-povidone (10:27:63). The prominent findings from this study elaborated that (a) Ternary ASD was able to provide high solubility of API in 0.1 N HCl, followed by an increased level of supersaturation after a pH shift to 6.8; (b) the longer supersaturation ability of ASD was primarily dependent on an addition of PVA no greater than 30% *w*/*w*; (c) an independent binary mixture of API: PVA (1:9) and API: co-povidone (1:9) failed to maintain efficient supersaturation in pH 6.8; (d) the miscibility of API with PVA was lower relative to co-povidone; however, the incorporation of PVA was deemed to be pivotal for inhibiting recrystallization together with co-Povidone.

Researchers from F. Postges et al. [81], worked on the poorly soluble drug celecoxib (BCS Class II) and investigated ASD made from HME using Eudragit L-100-55 and HPC SSL. Binary and ternary solid dispersions were prepared through HME using individual polymers and polymer combinations, respectively. The drug loading was at 10% *w*/*w* in the formulations. In this study, non-sink dissolution was conducted at pH 6.8. The author’s findings revealed a specific combination of two polymers found to be very critical to the extended phase of supersaturation. Either of the binary solid dispersions failed to provide efficient drug release comparative to ternary solid dispersion made with a 1:1 ratio of both tested polymers. Despite using a lower drug loading, the polymer composition and miscibility were the major driving force for the kinetic stabilization and precipitation inhibition. Therefore, this study exhibited the synergistic effect of the two tested polymers.

**Figure 1:**
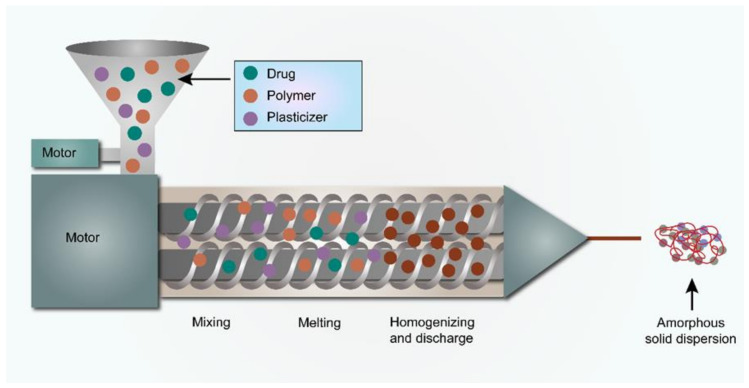
The schematic representation of the HME process.

The schematic representation of the hot-melt extrusion technology is shown in Figure 1.

#### 3.3.2. Polymer Combinations in Spray-Drying

In the case of ASD prepared from insoluble polymeric carriers, the drug release of poorly soluble API would be highly controlled through a diffusion-based phenomenon. Such systems contribute to the gradual occurrence of supersaturation; therefore, the risk associated with precipitation and crystal growth is very minimal. Conversely, these systems might be responsible for a limited release corresponding to the insoluble carrier. To improve the drug release from these systems, a hydrophilic polymeric carrier was used as a pore former by some researchers [82,83]. In a similar study by Everaerts et al. [84], Indomethacin ASD was manufactured using spray-drying, using Ethyl cellulose as a polymeric backbone and investigating two different grades of PVP (povidone), PVPK12 and PVP K25, respectively as water-soluble pore formers. Owing to the poor solubility of the drug in acidic media, the authors explored various amounts of the water-soluble carrier (PVP K12 and PVP K25) to evaluate its effect on improving drug release in acidic media. Their conclusions include the molecular weight of polymeric carriers, wettability of ASD, viscosity surrounding the particles, and molecular interactions that have a pivotal role to play in understanding the release mechanism. In addition, they noticed that, under 50% *w*/*w* PVP loading, the formulations containing PVP K12 outperformed the formulations with PVP K25 in acidic media, whereas, in pH 6.8, a similar release was observed irrespective of the type of PVP employed, due to the better solubility of API.

Ohyagi et al. [85] investigated the synergy between the polymers HPMC, methacrylic acid co-polymer Type A-Eudragit L100, methacrylic acid co-polymer Type B-Eudragit S in improving the solubility of griseofulvin (BCS class II) drug using SDD. Single-polymer and binary dispersions (in a 1:1 ratio) were made and dissolution testing was performed in the pH 7.4 buffer. The rate of dissolution from binary polymer SDD was significantly better than from single-polymer SDD. The improved dissolution rate is due to the reformation of hydrogen bonding, as analyzed by DSC and nuclear magnetic resonance (NMR) techniques. The SDD made with HPMC, Eudragit L100 enhanced the supersaturation of API. The authors established the technique of co-spray-drying two polymers, which improved intermolecular interaction, promoting the bioavailability of the poorly soluble drug.

**Figure 2:**
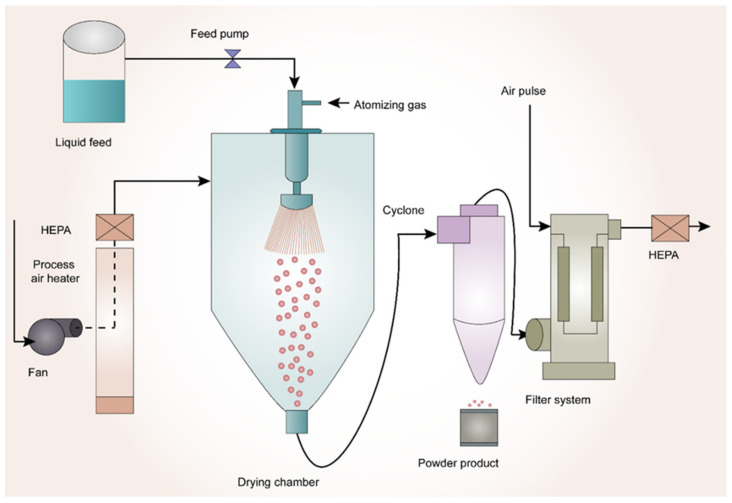
Schematic Representation of Spray-Drying Technology.

In a study by Rahman et al. [86], the authors tried to investigate the synergy between amphiphilic polymers soluplus^®^ and co-povidone to improve the supersaturation of griseofulvin. ASD was prepared by spray-drying technology. Drug-release testing was performed in purified water. SDD was made with 25% *w*/*w* drug loading. Ternary SDD composition with soluplus^®^ and co-povidone in the ratio of 5:1 was able to result in supersaturation of 220% in the initial 30 min, which was maintained over 3 h. Their research revealed that soluplus^®^ acted as a crystallization inhibitor in excess amounts relative to co-povidone acting as a wetting agent. The schematic representation of spray drying technology is shown in Figure 2.

### 3.4. Addition of Surfactants

#### 3.4.1. Addition of Surfactants in HME

Surfactants are amphiphilic molecules; when included in solid dispersions, they help to improve the wettability and reduce the contact angle to improve the solubility of crystalline API [87]. Thus, in the forthcoming sections, recent literature reports were discussed pertaining to the inclusion of surfactants and the role of the hydrophilic lipophilic balance (HLB) value of surfactants in the development of ASD through HME and spray-drying.

Kapourani et al. [88] manufactured ASD of BCS class IV drug Aprepitant using Soluplus^®^, HPC, and PVP using HME. The authors have examined the effect of surfactants like Vit E TPGS and Poloxamer P 407 in inhibiting drug crystal growth in solid dispersion. Among the polymers, evaluated PVP was found to be most effective based on API induction nucleation time in the pH 6.8 phosphate buffer. The ASD prepared with 10% *w*/*w* API and 10% *w*/*w* Vit E TPGS with PVP was found to be highly successful in inhibiting API recrystallization, due to enhanced miscibility and intermolecular interaction.

Another study by Saboo et al. [89], studied the incorporation of surfactants in enhancing the drug release in ASD prepared by HME. ASDs were prepared by using co-povidone as the main polymeric matrix and Felodipine as a poorly soluble drug. Vitamin E TPGS was chosen as a surfactant and dissolution studies were carried out in pH 6.8 phosphate buffer. The milled extrudates from HME were compressed into a tablet and evaluated for true or intrinsic dissolution using wood’s dissolution apparatus. From their study, it was concluded that the drug loading can be increased to 45% *w*/*w* and the formation of a drug-rich layer around ASD can be inhibited by the inclusion of 10% TPGS.

For investigating the lack of complete release from ASDs, Siriwannakij et al. [90], prepared ritonavir solid dispersions with 20% *w*/*w* drug loading using HME. In their study, the authors included 10% *w*/*w* of surfactants like Poloxamer 407 and Span 20, respectively in preparation for solid dispersions. The resultant melt extrudates were tested for drug release in gastric fluid pH 2 and phosphate buffer of pH 6.8, respectively. The dissolution samples analyzed for particle size from each media were tested. Their key findings reported that the presence of Poloxamer 407 was able to maintain the supersaturation until 75 min in gastric fluid, followed by recrystallization, which can be confirmed by particle size analysis. The addition of span 85 resulted in the maintenance of super saturation until 2 h, along with possessing a narrow particle size range. Despite of possessing a higher-HLB-value of 18 for Poloxamer 407, it was not molecularly dispersed in comparison to span 85 with a low HLB value of 1.8. Therefore, the molecular dispersion ability was found to be critical for efficient super saturation.

#### 3.4.2. Addition of Surfactants in Spray Drying

In a study by Yang and co-workers [91], spray-dried dispersions of BCS class IV drug Apremilast were manufactured using co-povidone and Vitamin E TPGS. DSC and PXRD studies indicated that the API turned amorphous in SDD. Dissolution studies were performed in phosphate buffer pH 6.8. Furthermore, their research concluded that the inclusion of TPGS promoted in vitro dissolution, along with improving the extent of absorption in an in vivo study relative to the physical mixture and Pure API.

Indulkar et al. [92] studied the effect of surfactants SDS, Tween 80, Span 20, Span 85, VIT E TPGS at 5% *w*/*w* concentration in improving the release performance of SDD made using co-povidone with 30% *w*/*w* Ritonavir. The SDD was compressed into tablets and evaluated for intrinsic dissolution in woods apparatus using pH 6.8 buffer. Among the surfactants tested, Span 85 performed better in terms of release, kinetic stabilization, and smaller droplet size of colloidal samples from dissolution.

In a study by Yan et al. [93], the non-ionic surfactants Tween 80, Vitamin E TPGS, and anionic surfactants such as sodium lauryl sulfate(SLS) were investigated in the formation of in situ nano particles. ASDs was manufactured using NCE GDC-0334 with co-povidone and surfactants by spray-drying. Dissolution studies were carried out in fasted-state simulated intestinal fluid (FASSIF) pH 6.5 and, after subsequent sample collection, the size analysis was carried out using the dynamic light scattering (DLS) technique. The SDD manufactured with 20% *w*/*w* drug load and 5% *w*/*w* Tween 80 exhibited an enhanced physical stability of nanoparticles. This phenomenon translated into an increase in vivo absorption in a rat pharmacokinetic study.

#### 3.4.3. Using Polymeric Salts for Enhancing Solubility

Another advancement in the formulation development of ASDs includes the use of polymeric salts as carriers. The dissolution rate and supersaturation ability of ASDs greatly depend on the dissolution rate of polymeric carriers. This becomes noticeable when using ionizable polymers such as hydroxypropylmethylcellulose phthalate. Qi and Taylor studied the factors that influence the dissolution of the enteric polymer, hydroxypropylmethylcellulose phthalate on the performance of ASDs using miconazole as a model drug. The authors prepared two polymeric salts, hydroxypropylmethylcellulose phthalate—sodium (HPMCP-50-Na) and HP-50-tetrabutylammonium (PTBA) from hydroxypropylmethylcellulose phthalate (HP-50) using acid-base reaction and salt metathesis reaction, respectively. The protonated HP-50 and the two polymer salts were formulated into ASDs with miconazole as a model poorly soluble drug. It is observed that the drug release was 14 times faster from the polymeric salts as compared to protonated HP-50. One of the reasons for this increase in drug release using polymeric salts can be due to the pre-ionization of the polymeric carrier, which resulted in a higher extent and rate of hydration. This forms a more mobile gel layer and enhances the release of polymer chains from the matrix, resulting in a higher drug release. Another reason for an increase in drug release using polymeric salts depends on the maintenance of stable micro environmental pH at the solid–water interface, since no protons were generated during the hydration of polymeric salts. While the physical stability of ASDs prepared using polymeric salts have yet to be studied, the use of polymeric salts could be a potential formulation strategy in the preparation of ASDs with high drug release [94].

## 4. Recent Advancements in the Manufacturing of ASDs

In this section, newly explored techniques in manufacturing ASDs such as Kinetisol^®^, 3D printing, and electronanospinning were briefly discussed, with a focus on the process and respective case studies available in the literature.

### 4.1. Kinetisol^®^

**Figure 3:**
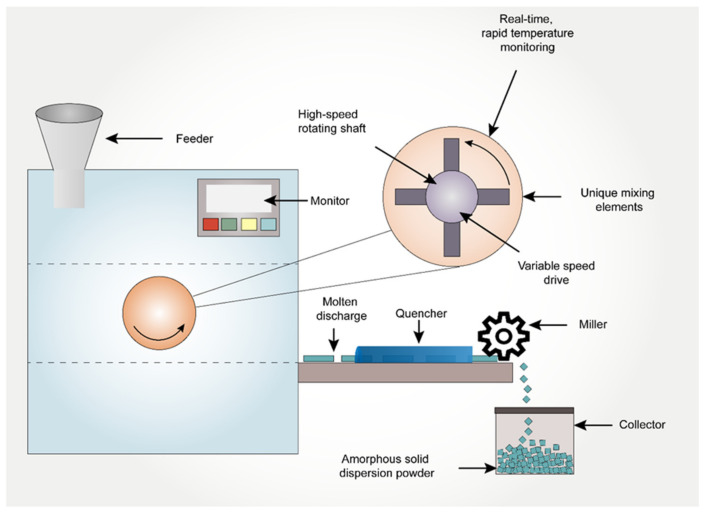
The schematic representation of Kinetisol^®^ process in preparation of amorphous solid dispersions.

Kinetisol^®^ is a novel technology that has been inherited from the plastic industry to the pharmaceutical field to enhance the solubility of poorly soluble API’s [95]. It is a fusion-based process that utilizes frictional and shear energies to rapidly transition drug-polymer blends into a molten state. Simultaneous to the transition to a molten state, Kinetisol^®^ rapidly and thoroughly mixes the API with its excipient carrier(s) at a molecular level to achieve a single-phase ASD system [96]. Polymers such as Carbopol, Eudragit, HPMC, HPMCAS, co-povidone, PVA, PVP, and Soluplus^®^ are widely used in Kinetisol^®^ processing [97]. First, the powdered blend is transferred into a chamber, which is then sealed. The processing parameters are pre-set using a computer module/software prior to the processing. The blades then rotate for a set time, during which the heat is generated due to the shear caused by the rotation of the blades. The powdered blend in between the rotating blades and the chamber wall is converted into a molten mass due to the heat generated by friction. The molten mass is then ejected to a quenching zone to form an amorphous flat disk. These quenched flat disks are further milled to fine granules of desired sizes. Finally, these milled granules are compressed into a tablet or filled in a capsule as a final drug product [95]. Total processing time within the chamber could be less than 20 seconds, and elevated temperatures are observed for typically less than 5 seconds before discharge and cooling. Since this technique uses non-heating elements to generate heat, the conversion of the API to its amorphous form takes place below the melting point of the compound, thus aiding in the formulation of thermolabile drugs [95]. The added benefit of this process relies on operating without torque limitation, thereby enabling the processing of viscous, high molecular weight, and high-melting-point compounds [29]. At the lab-scale, this process is designed to operate in batch mode, whereas, for commercial processing, there is an option to operate as a semi-continuous process, achieving a product throughput of as high as 1000 kg/h. The schematic representation of Kinetisol^®^ is indicated in Figure 3.

### 4.2. 3D Printing

**Figure 4:**
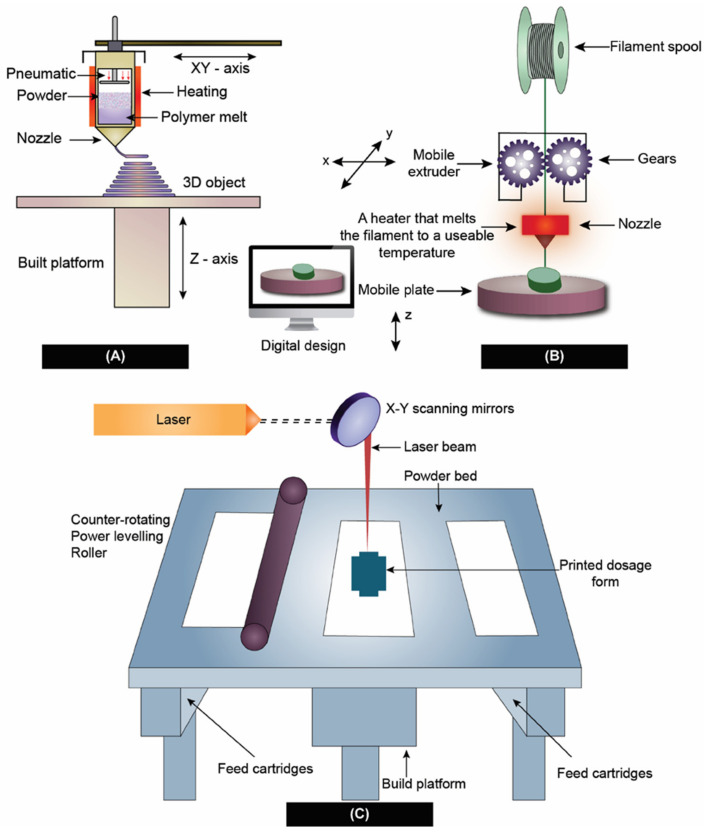
3D-printing techniques used in the preparation of ASD. (**A**) Direct powder extrusion, (**B**) Fused deposition modeling, and (**C**) Selective laser sintering.

3D-printed dosage forms have gained a lot of attention from researchers after the approval of the first 3D-printed oral formulation SPRITAM from Apprecia pharmaceuticals [31,98,99,100]. Pharmaceutical manufacturing in three-dimension arrangements (also known as three-dimensional printing or 3D printing or 3DP) bought a paradigm shift in formulation development of personalized dosage forms [101]. This cutting-edge technology uses additive manufacturing to transform 3D computer models into physical products [102]. Although, there are several different types of 3D-printing methods, the American Society for Testing and Materials (ASTM), has categorized them into seven categories namely, (i) Vat Photopolymerization, (ii) material jetting, (iii) binder jetting, (iv) material extrusion, (v) powder bed fusion, (vi) sheet lamination, and (vii) directed energy deposition [102]. However, the most widely used technologies for the preparation of ASDs reported in the literature include direct powder extrusion [103], SLS [104], 3D inkjet printing [105], and FDM [106]. These 3D-printing technologies are capable of making ASD just by the nature of virtue of their process. Among the various 3D-printing techniques mentioned above, SLS and FDM well-known in the preparation of ASDs. Therefore, in the following sections, we have attempted to emphasize selective laser-sintering SLS and FDM technologies for the preparation of ASDs. Figure 4 provides an outline of the preparation of ASDs using the 3D-printing techniques discussed in this manuscript.

#### 4.2.1. Selective Laser Sintering

Carl Deckard and Joe Beaman were considered pioneers in the development of the SLS in the early 1980s. When compared to other 3D-printing processes for pharmaceuticals, this technology offers numerous benefits in terms of there being no use of any solvents, rapid printability, no requirement for feed filaments, no addition of polymer liquid binder, and a lack of post-processing steps. After printing, printlets are readily available without any post-processing steps and curing. The essential prerequisite for this method includes the high thermal stability of formulation components at a higher temperature. Using the SLS printing technology, it is amenable to develop unique dosage forms such as amorphous solid dispersion and tailored medications for certain patient populations, such as children, the elderly, or people with disabilities [107].

By principle, SLS uses laser light to selectively heat powder particles, which ultimately leads to fusion and the formation of three-dimensional structures. After fusion, the structure will eventually solidify into a three-dimensional shape. The SLS system is composed of three primary elements, namely, a laser system (laser and scanner), a spreading platform, and a powder bed. By laser-sintering or melting between the particles, the material is heated to a temperature (below the melting point) that is high enough to induce fusion. Later, based on laser projection, the height of the powder bed alters, so that it focuses on the newly produced surface layer. Later, the surface of the powder bed lowers by an amount equal to the thickness of one layer; hence, the laser can be utilized to fuse another layer of powder. This procedure is repeated several times until the whole object is prepared. The finished product is extracted by hand or via sifting from the loose powder [108].

For the SLS process to deliver products with desired qualities, strict control over the parameters of the process is necessary. Many factors influence CQAs, such as the accuracy of stereolithography (STL) file conversion from computer-aided design (CAD) software, the slice into layers, machine resolution, the beam offset, and the thickness of the material shrinkage. The laser beam speed, laser watt, and the ratio of length to width are considered as important. Laser power [109,110], bed temperature [111,112,113], and layer thickness [114] are some of the most significant parameters influencing the CQAs of dosage forms during processing [115]. Thakkar and his co-workers [30] employed SLS-3D printing to develop ASDs containing Indomethacin, co-povidone, and Candurin^®^ blend. It was found that the speed of the laser, laser power, and intensity and duration of exposure have a profound effect on the conversion of the solid state of API to amorphous form. This study also concluded that the size of the drug particles also had a substantial influence on the apparent solubility of the drug, as well as the drug release. Figure 4C shows the method of preparation of ASD using SLS.

#### 4.2.2. Fused Deposition Modeling and Direct Powder Extrusion

Extrusion-based technologies such as FDM have received the most attention from researchers. After being patented in 1989 by the co-founder of Stratasys Scott Crump, FDM was made commercially available in the early 1990s [116]. The FDM method includes applying heat to a drug-loaded filament to extrude through a nozzle tip for depositing on a base plate, and then quickly cooling the extruded material in order to produce ASDs on the printing platform [117]. However, the requirement for HME to prepare drug dispersions, which increases the possibility of thermal drug degradation, is a significant disadvantage of FDM 3D printing [118,119]. The most significant advantages of FDM include, an inexpensive method, can be printed at a fast pace, and does not require complex equipment. However, the disadvantages of the FDM process include process parameter-dependent mechanical characteristics, poor surface finishing, and the fact that FDM printing materials are limited to thermoplastic polymers only [120,121,122]. However, numerous reports depicted its usage in dosage form development, such as that of Kissi et al. [123], used an FDM-based 3D-printing technique to formulate naproxen (NPA)-containing ASDs using co-povidone. Based on the results of the DSC and XRPD analyses, it was discovered that the ASDs within the 3D-printed tablets (20–30% *w*/*w* NPA) were amorphous and were stable for a 23-week duration at room temperature and 37% relative humidity. Similarly, Fanous et al. [124] developed a 3D-printed dosage form using FDM with an immediate drug release profile of a BCS class IV drug, Lumefantrine. In the preparation of the ASDs, Xylitol, Eudragit^®^ EPO, and maltodextrin were employed as a hydrophilic plasticizer, matrix former, and pore-forming agent, respectively. Raman mapping, a method with a high degree of sensitivity, was equipped to investigate the crystallinity of produced tablets and filaments. Lumefantrine tablets remained amorphous and showed sufficient stability for on-site production. Figure 4B shows the ASD preparation using FDM 3D-printing technology. Technique-wise, one of the drawbacks of FDM printing includes the requirement of HME to prepare drug-loaded filaments [118].

The limitations on the use of excipients and pharmaceutical drugs using FDM technology include the need to obtain filaments with the right mechanical and physical qualities [125]. In some cases, the filaments resulting from HME might end up being either too brittle or too soft, making them not amenable to 3D printing. Because of the drawbacks discussed earlier regarding HME, it would be beneficial to be able to bypass that stage. Recently, an updated version of FDM technology eliminated the use of HME by coupling extrusion with 3D printing. This process is called Direct Powder extrusion (DPE) [126]. In this process, a single screw extruder is used for the direct printing of pellets or powders by forcing them through a nozzle via an extrusion process, as shown in Figure 4C. This novel concept of utilizing 3D printing coupled with simultaneous extrusion enabled the conversion of crystalline API to an amorphous form during the process.

Goyanes et al. successfully 3D-printed Itraconazole printlets (3D-printed tablets), utilizing four varying HPC grades via a single-screw direct-powder-extruder 3D printer. The medication seems to be amorphous in formulations made with HPC–UL, and only partly amorphous in Formulations L, SL, and SSL, according to the results of PXRD and DSC. The improvement in solubility was found to be greater than when employing a different method (nanosuspension technology) with the same excipients and composition, which released only around 20% *w*/*w* of itraconazole. This demonstrates the effectiveness of the melting process in producing solid amorphous dispersions and supports the use of powder extrusion 3D-printing technology to improve the formulation’s solubility. Their key finding indicated that the molecular weight of the polymer may be reduced, which might boost the system’s wettability and increase drug release. Similarly, Guirales et al. also developed 3D-printed minitablets containing 20 mg of nifedipine combining hydroxypropyl cellulose and hydroxypropyl methyl cellulose acetate succinate in a single-step process. Direct powder extrusion can result in close contact between the drug and excipients, forming hydrogen bonds and thus resulting in ASDs confirmed with DSC and PXRD results.

This innovative single-step approach may be especially well-suited to preclinical research; it might revolutionize the development of amorphous solid dispersions as final formulations, and open a channel for flexibility when making patient-tailored dosage forms.

### 4.3. Electronanospinning

Despite existing since 1980, polymeric nanofibers gained recognition in the last decade in drug delivery for poorly soluble compounds. Drug-loaded polymeric fibers at the nano-size were found to be an innovative approach in the preparation of solid dispersions. This approach has gained a lot of attention from researchers due to its ability to convert the drug to an amorphous state with an increased surface area to enhance supersaturation and bioavailability. The electrospinning technique emerged as a versatile tool in the preparation of polymeric nanofibers as it is inexpensive and very rapid in the preparation of nano-size fibers via electrostatic forces [127,128,129].

**Figure 5:**
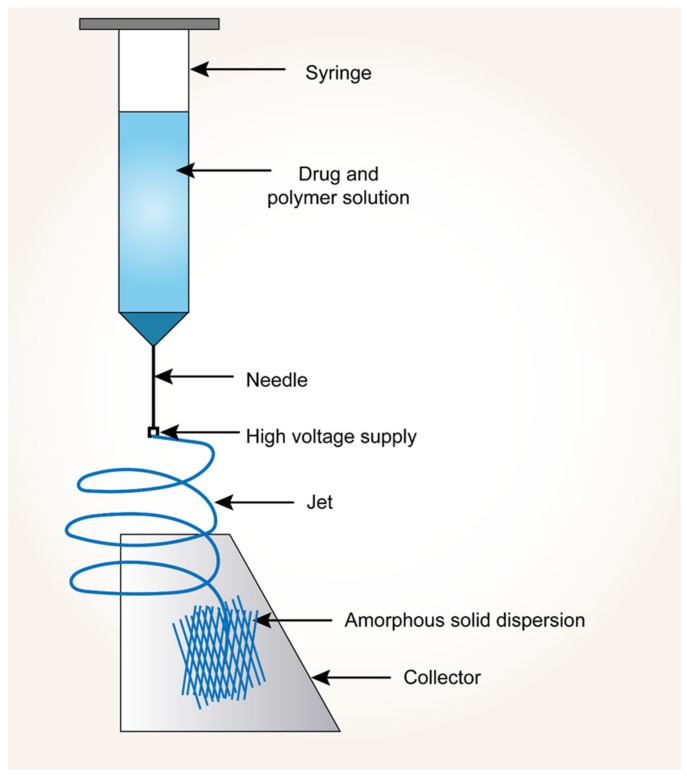
Process of electronanospinning approach.

In this technology, the drug and polymer are initially dissolved in a common volatile solvent. A high-energy potential difference (voltage) is applied between a flat-tipped needle spinneret (narrow gauge syringe needle) and a fiber collector (grounded). The travel and ejection of the drug–polymer solution take place with the applied high voltage. As the solution jet travels towards the grounded collector, the solvent evaporates and solidifies into nano-sized fibers. Due to the applied voltage to the drug-polymer solution, it creates repulsive forces between the charges and attractive forces between the charged solution and collector. In the electrospinning process, cone formation is found to be very critical, and to make this happen, the electrostatic force equals the surface tension of the liquid. Later, when this electrostatic force exceeds the surface tension, the solution breaks as a jet out of the cone. This accelerated jet stream stabilizes in the middle of the needle and collector [130,131]. Figure 5 shows the process of electronanospinning.

Verreck et al. [132] introduced the electrospun fibers of poorly soluble drug itraconazole using HPMC. In another study, Yi et al. [129] developed rapidly dissolving ibuprofen-loaded PVP nanofibers, which dissolved in <1 min. Nagy et al. [133] compared the drug dissolution behavior of spironolactone-Soluplus^®^ solid dispersions made by electrospinning to those extruded by HME; their key findings indicated that ASDs with the electrospun nanofibers demonstrated enhanced drug dissolution behavior relative to the HME-based solid dispersions.

## 5. Advances in Characterization Techniques

Analytical methods are necessary to precisely assess and describe the generation of crystalline counter parts for designing a stable amorphous solid dispersion. Current crystallinity quantification techniques were restricted to single polymorphic forms and quick crystallization kinetics in the threshold level of drug loadings. There are several traditional techniques to characterize ASDs, such as thermal analysis techniques (DSC, MDSC), thermogravimetric analysis; (TGA), microscopic/morphological techniques such as (PLM or hot-stage polarized light microscopy (HSPLM), PXRD, scanning electron microscopy (SEM), transmission electron microscopy (TEM), atomic force microscopy (AFM), spectroscopic techniques such as Infrared Spectroscopy (IR), Raman spectroscopy (RS), and fluorescence spectroscopy (FS). DSC and MDSC measure energy input associated with heating materials to detect thermal transitions such as T_g_, melting point, recrystallization, and polymorphic transformations [134]. TGA involves monitoring a sample’s weight as a function of temperature in a selected environment (such as air or nitrogen). It can be used to analyze the drug and polymer’s volatile components and assess their heat stability [135]. PLM is one of the best tools for finding traces of crystalline materials in ASDs. When examined with plane-polarized light and crossed polarizers, crystalline forms can be identified by their optical characteristics of birefringence [136]. In HSPLM, the sample is heated in a furnace in which the heating or cooling rate can be accurately controlled; therefore, in HSPLM heating is equipped with PLM [137]. PXRD is frequently employed to examine the crystallinity of samples and determine the presence of any crystalline matrices. In PXRD, characteristic diffractograms are based on the traces of crystallinity present in the tested sample. Using this technique, amorphous materials exhibit a halo pattern due to the lack of long-range order [138]. FS can help to understand the kinetics involved in the matrices of ASDs at high-humidity conditions and during dissolution. This technique detects the fluorescence emitted from a substance when excited by UV-visible radiation [139]. SEM is used to study the morphological changes in ASD samples after dissolution or a physical stability study. This technique uses a monochromatic electron beam to examine the surface and near-surface region of materials at a greater magnification and resolution [140]. TEM, on the other hand, is a very sensitive and effective method that can generate both real-space pictures and electron diffraction patterns to detect crystalline matrices in the samples tested [37,141]. AFM is used to detect the phase-separated regions of ASDs, when present in low quantities. It measures the height changes brought on by the contact between solid material and the sample. Different colors can be allocated in accordance with various heights, by processing these changes. It is mostly used to analyze surface topography (molecular surface roughness) of ASDs in the nanoscale range [142]. Drug–polymer interactions in ASDs can also be determined by tracking changes in peak location or shape using IR spectroscopy (changes in the dipole moment) and Raman spectroscopy (changes in the polarizability of a molecule) [143,144]. In this section, the state-of-the-art updated characterization techniques are discussed. Based on the literature, we have emphasized the most widely used techniques: terahertz spectroscopy, Dielectric spectroscopy, and X-ray microcomputed tomography. Along with the above-mentioned recent characterization techniques, we attempted to discuss the role of conventional methods and their application from the recent literature findings in Table 2.

### 5.1. Tetrahertz Spectroscopy

**Figure 6:**
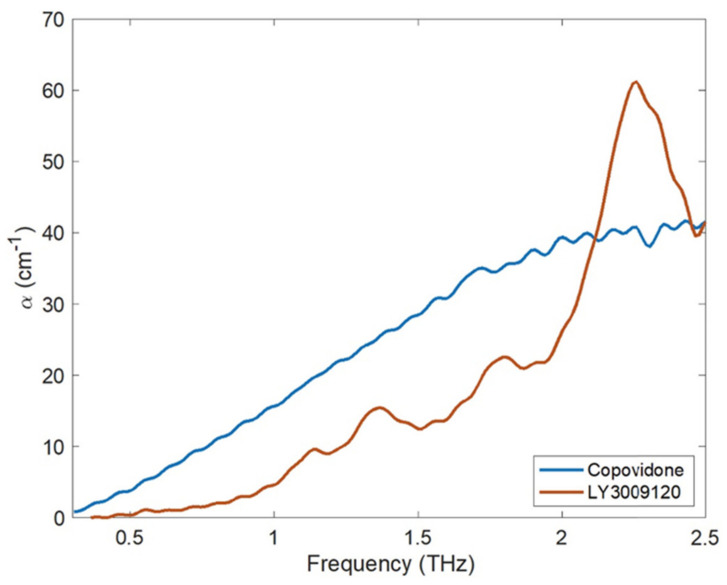
The THz absorption coefficient of co-povidone (amorphous) and LY3009120 as measured using THz-TD. Adapted from [159] under Creative Commons Attribution (CC BY) license (https://creativecommons.org/licenses/by/4.0/).

Terahertz (THz) spectroscopy and imaging have quickly emerged as a flexible analytical method in recent times. With a broadly defined frequency range of from 100 GHz to 10 THz, the terahertz portion of the electromagnetic (EM) spectrum falls between the infrared and microwave domains. The frequency range of most THz-TDS equipment, however, is around 0.1–4 THz (3–133 cm^−1^) [154]. It has qualities such as low energy, high directivity, a forceful penetration to most dielectric materials, and a huge capacity for both transmission and transport. The three core features of terahertz radiation include: (1) the capacity to measure low-energy intramolecular and intermolecular vibrations within and between molecules; (2) relatively low scattering losses owing to long wavelengths compared to typical particle sizes in pharmaceutical dosage forms; and (3) the capacity to penetrate a variety of polymeric and ceramic excipients due to low terahertz absorption. Terahertz pulsed spectroscopy (TPS) of amorphous materials exhibits no distinguishable spectral bands because TPS pertains to the intermolecular vibrations within the lattice structure rather than the intramolecular vibrations [155]. However, using TPS, the recrystallization phenomenon that could take place in an ASD may be monitored and validated [156]. The different spectrum shifts that occur with an increase in temperature provide vital information regarding relaxation and crystallization processes when measured in situ using temperature-dependent TPS [157]. In addition, TPS may be used in the process of determining the onset and intensity of molecular mobility, which is the fundamental process behind the crystallization of amorphous medicines [158]. Davis Jr. et al. [159] developed ASDs containing API LY3009120 using the Kinetisol^®^ process and evaluated the molecular interactions using terahertz spectroscopy. The crystalline form of LY3009120 and co-povidone’s THz absorption spectra is shown in Figure 6. The authors reported that Kinetisol^®^ formulations produced a miscible system that confirmed that formulations reduced the absorption coefficient compared to the physical combination.

### 5.2. Dielectric Spectroscopy

Dielectric spectroscopy (DS), also known as impedance spectroscopy and electrochemical impedance spectroscopy, is often used to investigate the response of a sample that has been exposed to an applied electric field of a constant or varying frequency [160]. Recent years have witnessed a surge in the utilization of dielectric methods as experimental approaches to study relaxation processes in amorphous pharmaceutical systems [43]. Since both the cooperative and non-cooperative motion of drug molecules may be determined from this technique, dielectric spectroscopy has been utilized to detect the time scale of intramolecular and molecular motion. Similarly, researchers such as Pacult et al. [161] used dielectric spectroscopy to explore the relaxation behavior of co-amorphous systems containing flutamide and bicalutamide. The authors reported that the developed formulations did not exhibit any signs of crystallization after being subjected to a super-cooled liquid condition. Another research group, Anusaya et al. [162], investigated the influence of cross-linkers on the stability and molecular mobility of ASDs containing Ketoconazole using DS. The longer α-relaxation time revealed that the molecular mobility decreases as the concentration of cross-linkers in the formulation increases.

### 5.3. X-ray Micro-Computed Tomography

**Figure 7:**
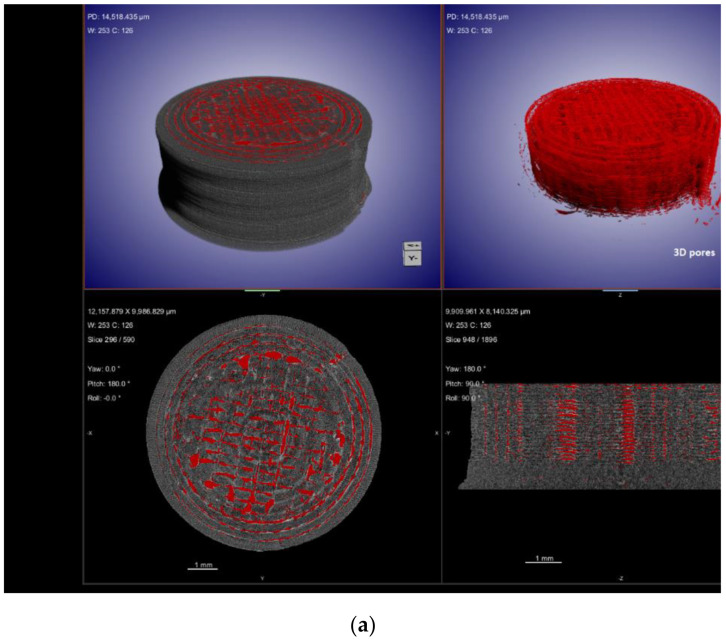

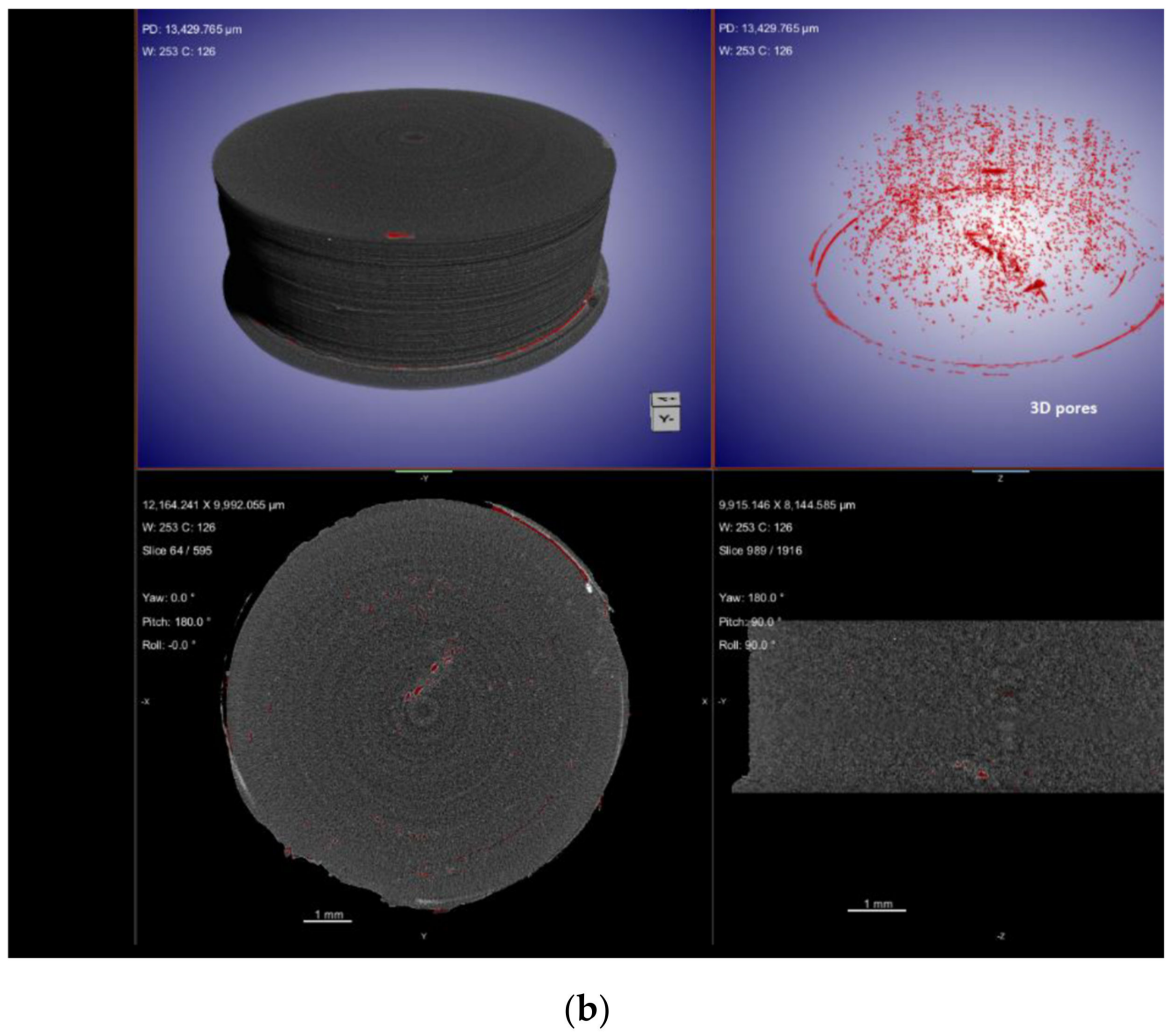
XµCT images showing material deposition and 3D pore distribution (red) in 3D-printed tablets containing (**a**) 20% *w*/*w* NAP and (**b**) 30% *w*/*w* NAP. Adapted from [123] under Creative Commons Attribution (CC BY) license, (https://creativecommons.org/licenses/by/4.0/).

X-ray computed tomography (XµCT) is capable of offering information on the interior structure of materials in a non-destructive manner on length scales ranging from meters to tens of nanometers. Using the penetrating power of X-rays, it obtains a sequence of two-dimensional radiographs of the element viewed from a variety of angles, sometimes referred to as XµCT Scan [163]. From these 2D projections (radiographs) of the object, a computed reconstruction algorithm is employed to build a stack of cross-sectional slices. This procedure generates a computerized 3D greyscale depiction (commonly referred to as a tomogram) of the object’s underlying structure. For a better view of 3D morphology, this may be statistically analysed and virtually dissected in any direction. Additionally, particular constituents can be digitally color-coded or rendered clear to obtain the desired effect. Imaging using XµCT has a number of benefits compared to other methods, the most important of which is that it is non-destructive. In the process of ASD characterization, XµCT has been utilized to view and quantify the structure of spray-drying particles. This includes the wall thickness and interior structures of the particles [164]. In addition, XµCT has been employed as a quantitative approach to assess the drug-phase separation in patches that have been manufactured via HME and injection molding [165]. Kissi et al. [123] prepared ASDs of Naproxen (NAP) with varying drug loads (10–30% *w*/*w*) using 3D-printing technology. According to the results of the XµCT, the nozzle may not deposit material in all areas during the 3D-printing of tablets containing 20% *w*/*w* of drug, as specified in the design file, whereas only a few air voids were produced for the 30% *w*/*w* naproxen tablet, resulting in a denser structure, as observed in Figure 7. The absence of a printing wall or infill density pattern indicates that the structure is dense, suggesting good material flow, which is determined with the aid of this advanced XµCT technique.

Approaches to thermal analysis and spectroscopic analysis evolved as an essential component of the characterization process for ASDs. These measures allow for the acquisition of a wide variety of useful information, such as the assessment of a substance molecular mobility, crystallization behavior, and system miscibility. The characterization of ASDs has received a lot of attention and research in the last ten years, which has led to a lot of development. Considering the increasing use of amorphous formulation strategies to deal with poor aqueous solubility, these novel techniques, and the information gleaned from them are likely to become more sophisticated, further enhancing our understanding of the fundamental properties of these formulations.

## 6. Computational Models for Stability Predictions

At present, determining the physical stability of ASDs by trial-and-error would require at least three–six months. If this time-consuming process fails, it is important to try again. Furthermore, the process behind the physical stability of solid dispersions is not yet fully understood [166]. In recent years, a number of hypotheses concerning the stability of solid dispersions have been debated, including those concerning the solubility parameters and the T_g_ prediction model [167]. These theoretical models require a substantial quantity of physicochemical information on each constituent, as well as a great deal of expert-level expertise. In addition to this, the power of these models to make predictions was relatively restricted because of the uncontrolled inaccuracy caused by the mathematical postulate [168].

Another useful tool is molecular modelling, which combines theoretical and computational methods to simulate the behavior of molecules at the atomic level; this type of modelling is known as molecular simulation [169]. The use of machine learning has the potential to make data-driven decision-making easier, to speed up processes, and minimize the number of times that procedures fail. Several researchers are employing these intelligent systems to forecast the physical stability of ASDs. Computational techniques make it possible to obtain a deeper knowledge of ASD phenomena, particularly when used in tandem to supplement and inform trials, as well as to aid in the construction of prediction models for the pragmatic formulation design process. Molecular modeling and simulation approaches help to clarify crucial stabilizing intermolecular interactions between API and carrier, estimate solubility parameters, simulate ASD formation and dissolution procedures, and create descriptors for quantitative structure-property connections (QSPR) [170].

Lee et al. [171] explored deep learning techniques for predicting the stability of ASDs which is time-consuming and expensive, thus, offering a brand-new prediction model architecture. Correspondingly Han et al. [168] also investigated a novel machine learning method to overcome the conventional ASDs stability prediction approach. In this study, the RF algorithm effectively constructed the prediction model with 646 formulation data and achieved an accuracy of 82% *w*/*w* for solid dispersion physical stability. This prediction model also makes clear the significance of each formulation element, which has a substantial positive impact on the design of solid dispersion formulations.

The combined theoretical, modelling, experimental, and data-driven AI technique may be used in the formulation production of future alternative dosage forms. This is a revolutionary strategy to increase the effectiveness and precision of developing solid dispersion formulations to construct an intelligent system for the stability predictions using machine-learning techniques.

## 7. Conclusions and Future Perspectives

Over the decades, ASDs have emerged as one of the most promising approaches for the solubility enhancement of poorly water-soluble drugs. The use of high-throughput screening techniques combined with a better understanding of the physicochemical properties of NCEs resulted in advances in preformulation strategies for ASDs. The knowledge acquired during preformulation studies helps to understand the potential challenges in formulating ASDs, such as recrystallization during stability, increased pill burden (based on required dose), or precipitation during in vitro dissolution. These challenges were mitigated using the recent developments in formulation strategies, such as the use of polymeric combinations, and the use of surfactants or polymeric salts. In addition to the preformulation and formulation advances, various recent technological advances have emerged over the decades, which have led to the commercial capability of formulating ASDs. These technologies also have the potential for continuous manufacturing of ASDs, which still needs to be explored to make them viable for commercial setup. The recent advances in the field of engineering resulted in improved characterization techniques that help to understand the morphological aspects of ASDs at a submicron level, which was not possible earlier. Some of these advanced characterization techniques (Raman imaging) have the potential to be a process analytical tools (PAT) tool in the continuous manufacturing of ASDs. In the current review, an attempt was made to summarize the prominent and recent advances related to the formulation of ASDs. There is still a lot of research ongoing to better understand ASDs and overcome the associated challenges.

## Figures and Tables

**Table 1:** Examples of FDA-approved amorphous solid dispersions products. Adapted from [18] under Creative Commons Attribution (CC BY-NC-ND 4.0) license, (https://creativecommons.org/licenses/by/4.0/).

Trade Name	Chemical Name	BCS Class	Manufacturing Technique	Polymers Used	Company	Year of Approval
Cesamet^®^	Nabilone	II	Solvent evaporation	Povidone	Meda Pharmaceuticals	1985
Isoptin SR	Verapamil HCl	II	Melt extrusion	Hypromellose	Ranbaxy Laboratories	1987
Sporanox	Itraconazole	II	Fluid bed bead layering	Hypromellose, Polyethylene glycol	Janssen	1992
Prograf	Tacrolimus	II	Spray drying	Hypromellose,	Astellas Pharma	1994
NuvaRing	Etonogestrel/Ethinyl Estradiol	II	Melt extrusion	Ethylene vinylacetate copolymer	Merck	2001
Kaletra	Lopinavir/Ritonavir	IV/IV	Melt extrusion	Co-povidone,	AbbVie	2007
Intelence	Etravirine	IV	Spray drying	Hypromellose	Janssen	2008
Modigraf	Tacrolimus	II	Spray drying	Hypromellose	Astellas Pharma	2009
Zortress	Everolimus	III	Spray drying	Hypromellose	Novartis	2010
Norvir Tablet	Ritonavir	IV	Melt extrusion	Co-povidone	AbbVie	2010
Onmel	Itraconazole	II	Melt extrusion	Hypromellose	Merz Pharma	2010
Incivek	Telaprevir	II	Spray drying	Hypromellose acetate succinate	Vertex	2011
Zelboraf	Vemurafenib	IV	Solvent/anti-solvent precipitation	Hypromellose	Roche	2011
Kalydeco	Ivacaftor	II	Spray drying	Hypromellose acetate succinate	Vertex	2012
Noxafil	Posaconazole	II	Melt extrusion	Hypromellose acetate succinate	Merck	2013
Harvoni	Ledipasvir/Sofosbuvir	II/III	Spray drying	Co-povidone	Gilead Sciences	2014
ViekiraXR™	Dasabuvir/Ombitasvir/Paritaprevir/Ritonavir	II/IV/IV/IV	Melt extrusion	Co-povidone	AbbVie	2014
Epclusa	Sofosbuvir/Velpatasvir	III/IV	Spray drying	Co-povidone	Gilead Sciences	2016
Orkambi	Lumacaftor/Ivacaftor	II/II	Spray drying	Hypromellose acetate succinate, Povidone	Vertex	2016
Venclexta	Venetoclax	IV	Melt extrusion	Co-povidone	AbbVie	2016
Zepatier	Elbasvir/Grazoprevir	II/II	Spray drying	Vitamin E polyethylene glycol succinate, Co-povidone, Hypromellose	Merck	2016
Stivarga	Regorafenib	II	Solvent Evaporation	Povidone	Bayer	2017
Mavyret™	Glecaprevir/Pibrentasvir	IV/IV	Melt extrusion	Hypromellose, Co-povidone	AbbVie	2017
Lynparza	Olaparib	IV	Melt extrusion	Co-povidone	AstraZeneca	2018
Erleada	Apalutamide	II	Spray drying	Hypromellose acetate succinate	Janssen	2018
Trikafta	Elexacaftor (Crystalline)/Ivacaftor/Tezacaftor	II or IV	Spray drying	Hypromellose, Hypromellose acetate succinate	Vertex	2019
Symdeko	Tezacaftor/Ivacaftor and Ivacaftor	II/II or IV	Spray drying	Hypromellose, Hypromellose acetate succinate	Vertex	2019
Braftovi	Encorafenib	II	Melt extrusion	Co-povidone, Poloxamer 188	Pfizer	2020
Oriahnn™	Elagolix/estradiol/norethindrone acetate	III/II/NA	Melt extrusion	Co-povidone, Hypromellose	AbbVie	2020

**Table 2:** List of conventional analytical techniques used in the characterization of ASDs.

Technique	Key Characteristics	Advantages	Limitations	Applications	Ref
Differential Scanning Calorimetry	Determination of melting point, glass transition temperature, heat capacity, drug and polymer interactions, determine the degree of crystallinity, and drug crystallization tendency; identify crystalline and amorphous state and molecular mobility.	Suitable for measuring melting; small sample size; easy experimental conditions; cost-effective and quick	Destructive, heat capacity measurement is less sensitive, no knowledge of the nature of the thermal events, and simultaneous thermal events that overlap cannot be resolved.	Solomon et al. utilized DSC in order to estimate the distribution of Felodipine in ternary amorphous dispersions containing Soluplus^®^ and silica. They computed theoretical T_g_ of binary Felodipine-soluplus^®^ combinations using the Gordon–Taylor equation in their research and compared them to T_g_ obtained empirically. They concluded that DSC results in a negative deviation in T_g_, confirming stronger hydrogen bonding interactions between drug and polymer.	[145]
Modulated Differential Scanning Calorimetry	To assess the crystallization tendency of active ingredients, glass transition temperature, knowledge about miscibility with polymers, studying degree and level of crystallinity, crystal growth rate, and molecular mobility (e.g., structural relaxation, viscosity)	Separation of overlapping and complex thermal events, improved sensitivity in heat capacity measurement in comparison to conventional DSC.	Requirement of well-planned experiments, experimentation is very conditional dependent, Melting: challenging interpretation, unreliable mDSC measurement	Zhang et al. performed in silico screening first and further various prototype formulations of apremilast ASDs via spray drying were developed. In this study, the author utilized mDSC to study the miscibility of the drug with polymer, measure T_g_, and evaluate the stability of ASDs under stress conditions. It was observed via mDSC analysis that immiscible systems exhibit instability after being stored under stressful conditions and showed multiple T_g_ in comparison to single T_g_ obtained from stable ASDs.	[146]
Thermogravimetric analysis	Thermal stability, study evaporation profile of feed solution in spray-drying, chemical identification of volatile compounds released from samples	Limited sample size, little sample preparation required, simple to use	Destructive, unable to determine the chemical composition	Yu et al. studied the role of neutral and acidic polymers in the physical and chemical stability of the developed ASDs of carbamazepine. The DSC and TGA analysis revealed that the prepared ASDs are physically stable owing to the formation of strong intermolecular bonds; however, acidic polymers provide an acidic microenvironment, making the developed ASDs susceptible to chemical degradation.	[147]
Fourier Transformation Infrared technique (FTIR)	Molecular interactions between drug and polymer, polymorph characterization, phase separation, crystalline and amorphous identification	Quantitative analysis, small sample requirement, non-destructive	Moisture present and less precise findings	Bhanderi et al. confirmed the development of ASDs of griseofulvin and hypromellose acetate succinate along with surfactant employing FTIR. Authors concluded that the peak positions and broadening in the developed ASDs were unaffected by the surfactant presence, indicating that the polarity around the aforementioned groups was unaffected.	[148]
Powder X-ray diffraction	Polymorphs screening, detects crystallinity degree, amorphous detection, and drug–polymer miscibility, studies recrystallization behavior, the microstructure of ASDs	The sample size required is small, analysis is simple, qualitative and quantitative, non-destructive	Relatively less sensitive (>5% crystallinity) than DSC, TEM and PLM, details on the chemical structure is provided, which can otherwise be obtained from nuclear mass resonance spectroscopy, infrared spectroscopy and mass spectrometry.	Bhujbal et al. studied Lumefantrine ASDs physical stability and dissolution profile and observed that (a) polymer and (b) drug-to-polymer ratio had a significant effect. Eudragit L 100 showed a crystalline drug peak even at lower drug concentration in PXRD analysis whereas HPMC Phthalate and HPMC AS showed better physical stability and miscibility with the drug.	[149]
Polarized Light Microscopy	Polymorphic transition, detect crystallinity (birefringence) and amorphous, crystal shape and size, crystallization route	Non-destructive, simple to use, smaller sample size, reproducible	It is not appropriate for agglomerates, semi-quantitative, and sample recovery is quite laborious.	Moritani et al. prepared tranilast-loaded ASDs for the treatment of inflammatory bowel disorders. There was no discernible birefringence in the PLM picture of ASDs/tranilast, whereas crystalline drug showed considerable birefringence. This result was also in agreement with DSC.	[150]
Atomic force microscopy	Visualises molecular mobility, mixture-specific separation rates, drug–polymer miscibility, and bulk and surface dynamics. These variables have a fundamental and integral role in predicting the long-term stability of an ASD.	High resolution up to 1 nm Small sample size, Detect repeated lattice	Expensive, Lengthy scan duration that may cause sample thermal drift, sample preparation is necessary	Zhao et al. developed quaternary enteric ASDs of erythromycin utilizing HME. Raman and AFM have taken advantage of the fact that the bulk of the drug dispersed in the PVP/VA64 matrix (co-povidone), and that the nanometre-sized drug–polymer system confined within the enteric continuous phase to form a solid emulsion-like structure.	[151]
Scanning electron microscopy	Analyze particle size, morphology, and surface characteristics of formulation, identifies present of drug crystal, chemical distribution map	Low sample size and high resolution	Necessitates sample preparation (coating and vacuum setting), costly equipment, huge device, and requires housing in a location free from any potential electric, magnetic, or vibration interference	Spray-drying was carried out to prepare ASD formulations of GDC-0334 in PVP/VA 64 (co-povidone) at different drug loadings (20, 30, 40, and 60 percent, *w*/*w*), with or without 5% *w*/*w* surfactants (Tween 80, SLS, or Vit-E TPGS 1000) by Yen and his coworkers. All four ASD formulations with 5% *w*/*w* Tween 80 as constant and varied drug-loading (20–60% *w*/*w*) displayed a collapsed spherical shape made up of small, dense particles with sizes between 2 and 3 µm.	[93]
Solid-state nuclear magnetic resonance (SSNMR)	Detect crystallinity degree, amorphous identification, drug and polymer interaction, drug and polymer miscibility, molecular mobility	Limited sample size, qualitative and quantitative, easy to prepare the sample, non-destructive	Possibility of recrystallization during analysis, relatively expensive, quantification challenges caused by chemical noise and signal overlaps, longer analysis time	Jarrells et al. measured and compared ASDs crystallinity using the SSNMR technique for nifedipine and polyvinylpyrrolidone drug–polymer system. Authors successfully were able to distinguish residual crystals and crystals formed during storage via SSNMR.	[152]
Raman Spectroscopy	Can be applied to study the dissolution behavior in aqueous conditions, and gives information about drug–drug and drug–polymer interaction, drug–polymer miscibility, and phase separation.	Quantitative detection, small sample size, unaffected by water, ability to penetrate glass containers	Sample heating with powerful laser radiation can harm the sample, sample fluorescence and photodegradation, requires sensitive and highly specialised instruments for detection.	Paisana et al. formulated ASDs of itraconazole employing HPMC-acetate succinate medium grade (HPMC-AS M). Author evaluated ASDs suspended in FaSSIF media after 240 min, Raman examination of API: HPMC-AS M (35:65) particles confirmed a faster polymer dissolution rate and associated surface API enrichment.	[153]
Transmission electron microscopy	To detect crystalline substances in ASDs, can generate both real-space pictures and electron diffraction patterns.	Smaller sample quantity, quantitative, high spatial resolution imaging, ability to detect crystallinity degree	Certain samples may be damaged by electron beams; tedious sample preparation	Sari et al. prepared ASD of felodipine and polyvinylpyrrolidone/vinyl acetate copolymer (co-povidone) employing the HME technique. PXRD, DSC, and FTIR revealed no evidence of residual crystallinity. However, of the 55 investigated particles, two places with crystals at the edges of milled particles were found using TEM.	[37]
